# Defining the Inflammatory Plasma Proteome in Pediatric Hodgkin Lymphoma

**DOI:** 10.3390/cancers12123603

**Published:** 2020-12-02

**Authors:** Jennifer E. Agrusa, Brooks P. Scull, Harshal A. Abhyankar, Howard Lin, Nmazuo W. Ozuah, Rikhia Chakraborty, Olive S. Eckstein, Nitya Gulati, Elmoataz Abdel Fattah, Nader K. El-Mallawany, Rayne H. Rouce, ZoAnn E. Dreyer, Julienne Brackett, Judith F. Margolin, Joseph Lubega, Terzah M. Horton, Catherine M. Bollard, M. Monica Gramatges, Kala Y. Kamdar, Kenneth L. McClain, Tsz-Kwong Man, Carl E. Allen

**Affiliations:** 1 Department of Pediatrics, Baylor College of Medicine, Texas Children’s Cancer and Hematology Centers, Houston, TX 77030, USA; bpscull@texaschildrens.org (B.P.S.); haabhyan@texaschildrens.org (H.A.A.); howard.lin@bcm.edu (H.L.); nwozuah@texaschildrens.org (N.W.O.); rxchakra@texaschildrens.org (R.C.); oseckste@texaschildrens.org (O.S.E.); nxgulati@texaschildrens.org (N.G.); elmoataz.abdelfattah@bcm.edu (E.A.F.); nkelmall@texaschildrens.org (N.K.E.-M.); rhrouce@texaschildrens.org (R.H.R.); zedreyer@texaschildrens.org (Z.E.D.); jxbracke@texaschildrens.org (J.B.); jfmargol@texaschildrens.org (J.F.M.); lubega@bcm.edu (J.L.); tmhorton@texaschildrens.org (T.M.H.); gramatge@bcm.edu (M.M.G.); kykamdar@texaschildrens.org (K.Y.K.); klmcclai@texaschildrens.org (K.L.M.); ctman@texaschildrens.org (T.-K.M.); 2 Center for Cancer and Immunology Research, Children’s National Health System and The George Washington University, Washington, DC 20010, USA; cbollard@childrensnational.org

**Keywords:** Hodgkin lymphoma, childhood hematological malignancies, immunology, chemokines, cytokines

## Abstract

**Simple Summary:**

Hodgkin lymphoma (HL) is a common type of cancer that is characterized by rare, malignant cells among an inflammatory microenvironment. Specific systemic, inflammatory plasma proteins have demonstrated prognostic significance in adult HL; however, systemic inflammation has not been well-characterized in childhood HL. The aim of our study was to better define the inflammatory pre-therapy plasma proteome and identify plasma proteins associated with clinical features of childhood HL. We measured plasma concentrations of 135 proteins in 56 pediatric subjects with newly diagnosed HL and 47 healthy pediatric controls. We found that the plasma protein profile was distinct from controls, and unique proteins were associated with high-risk disease (IL-10, TNF-α, IFN-γ, IL-8), slow early therapy response (CCL13, IFN-λ1, IL-8), and relapse (TNFSF10). These proteins could be used to improve risk stratification, and thus optimize outcomes and minimize unnecessary toxic exposures for those with childhood HL.

**Abstract:**

Hodgkin lymphoma (HL) histopathology is characterized by rare malignant Reed–Sternberg cells among an inflammatory infiltrate. We hypothesized that characteristics of inflammation in pediatric HL lesions would be reflected by the levels of inflammatory cytokines or chemokines in pre-therapy plasma of children with HL. The study objectives were to better define the inflammatory pre-therapy plasma proteome and identify plasma biomarkers associated with extent of disease and clinical outcomes in pediatric HL. Pre-therapy plasma samples were obtained from pediatric subjects with newly diagnosed HL and healthy pediatric controls. Plasma concentrations of 135 cytokines/chemokines were measured with the Luminex platform. Associations between protein concentration and disease characteristics were determined using multivariate permutation tests with false discovery control. Fifty-six subjects with HL (mean age: 13 years, range 3–18) and 47 controls were analyzed. The cytokine/chemokine profiles of subjects with HL were distinct from controls, and unique cytokines/chemokines were associated with high-risk disease (IL-10, TNF-α, IFN-γ, IL-8) and slow early response (CCL13, IFN-λ1, IL-8). TNFSF10 was significantly elevated among those who ultimately relapsed and was significantly associated with worse event-free survival. These biomarkers could be incorporated into biologically based risk stratification to optimize outcomes and minimize toxicities in pediatric HL.

## 1. Introduction

Hodgkin lymphoma (HL) is a common childhood cancer characterized by an inflammatory microenvironment that contributes to disease pathogenesis. Malignant Hodgkin Reed–Sternberg (HRS) cells make up less than 1% of the tumor, and the remainder is composed of inflammatory cells [1]. Inflammatory mediators, including C-C motif chemokine 17 (CCL17, also known as thymus and activation regulated chemokine (TARC)), soluble CD163 (sCD163), tumor necrosis factor receptor superfamily member 8 (also known as soluble CD30 (sCD30)), interleukin-6 (IL-6), soluble IL-2 receptor alpha (IL-2Rα, also known as sCD25) and soluble Galectin-1 (sGal-1), are prognostic in adult HL [2,3,4,5,6,7].

Few studies have investigated the prognostic implications of the plasma proteome in pediatric HL (Table 1). Some have associated elevated concentrations of soluble intracellular adhesion molecule-1 (sICAM-1) and vascular endothelial growth factor receptor 1 (VEGF-1) with poor outcomes [8,9,10,11]. However, these findings have not been consistently reproduced. The biological and clinical significance of the inflammatory infiltrate components in pediatric HL remains largely undefined [12].

Treatment of HL includes intensive empiric therapies that produce relatively high cure rates; however, current treatment regimens also confer risk for therapy-related toxicities [13,14,15], especially for subgroups with slower therapy response or relapsed/refractory disease who require augmented therapy and hematopoietic stem cell transplant [16]. To reduce unnecessary exposures and identify those at higher risk for treatment failure, there have been efforts to improve the risk stratification system in HL. Improving methods for identifying patients at highest risk for treatment failure who warrant intensified therapy, and lower risk patients who could achieve cure with reduced intensity therapy, is a key unmet need in the clinical management of adult HL [17]. In comparison to adult HL, there are limited studies evaluating potential biomarkers that may inform risk stratification in pediatric HL, although the costs of ineffective or excessive therapy are greater. We hypothesize that the cytokine/chemokine profile of pediatric HL may inform clinical outcomes. The objective of this study is to better define the inflammatory pre-therapy plasma proteome in a cohort of pediatric patients with HL and to identify candidate plasma biomarkers associated with extent of disease, early response to therapy, and treatment failure. These biomarkers may be incorporated into existing clinical stratification to improve the treatment and outcome of the patients.

**Table 1:** Proteins Evaluated in Study Cohort Previously Reported in Adult or Pediatric HL.

Protein	Alternative Protein Name(s)	Significance in Study Cohort	Previously Reported in HL	Previously Reported Significance
C-C motif chemokine 17 (CCL17)	CC chemokine TARC, Small-inducible cytokine A17, Thymus and activation-regulated chemokine (TARC)	Elevated in HL vs. Controls	Adult HL	Pretreatment levels were associated with clinical risk factors, therapy response [6].
			Adult HL	Disease response marker [2].
			Adult HL	Increased levels correlated with Ann Arbor stage in NS HL; majority showed decreased levels after treatment [18].
			Adult HL	Accurately reflects disease activity, correlates with treatment response [5].
			Adult HL	Baseline levels correlate with tumor burden, serial levels correlate with therapy response [19].
			Adult HL	Change in TARC is not prognostic [20].
C-C motif chemokine 22 (CCL22)	Macrophage-derived chemokine (MDC), MDC (1–69), CC chemokine STCP-1, Small-inducible cytokine A22, Stimulated T-cell chemotactic protein 1	None	Adult HL	Increased levels correlated with Ann Arbor stage in nodular sclerosing HL. Majority showed decreased levels after treatment [18].
Fibroblast growth factor 2 (FGF-2)	Basic fibroblast growth factor (bFGF), Heparin-binding growth factor 2 (HBGF-2)	Elevated in HL vs. Controls	Adult HL	Cases with CD30+ cells carrying an FGF2+/SDC1+ immunophenotype had shortened survival [21].
Interleukin-1 receptor antagonist protein (IL-1RA)	None	None	Adult HL	High levels were independent poor prognosis factors (EFS, OS) [22].
Interleukin-2 (IL-2)	None	None	Pediatric HL	Very high levels were significantly associated with poor outcomes in HL [23].
Interleukin-6 (IL-6)	None	Elevated in HL vs. Controls	Adult HL	High levels were independent poor prognosis factors (OS, EFS) [22].
			Adult HL	Associated with increased relapse, poor survival, sCD30 and TARC levels [4].
			Adult HL	Useful for disease monitoring [24].
Interleukin-10 (IL-10)	None	Elevated in HL vs. Controls, associated with risk	Adult HL	Useful for disease monitoring [24].
			Adult HL	Associated with early treatment failure [25].
			Pediatric HL	Increased levels were associated with HL symptoms; pre-treatment levels were higher in non-responders (multiple diagnoses) [26].
Interleukin-12 (IL-12)	None	None	Pediatric HL	Pre-treatment IL-12 was lower in non-responders (multiple diagnoses) [26].
Interleukin-13 (IL-13)	None	None	Adult HL	Useful for disease monitoring [24].
Macrophage colony-stimulating factor 1 (M-CSF)	None	None	Adult HL	Increased levels correlate with bulky mediastinal disease, systemic symptoms [27].
Tumor Necrosis Factor Receptor (TNF receptor)	None	None	Adult HL	Associated with lower EFS/OS [22].
Vascular endothelial growth factor (VEGF)	None	Elevated in HL vs. Controls	Pediatric HL	Independent risk factor for treatment failure [10].
			Pediatric HL	Changes in levels correlate with treatment response [9].

Abbreviations: HL, Hodgkin lymphoma; NS, nodular sclerosing; EFS, event-free survival; OS, overall survival.

## 2. Results

### 2.1. Cohort Characteristics

Fifty-six pediatric subjects with HL and 47 pediatric healthy controls were included in this study (Tables S1 and S2). Demographic and clinical features of the HL cohort are shown in Table 2. Characteristics are typical of a pediatric HL cohort.

Forty-one subjects with low-risk/intermediate-risk (LR/IR) HL were treated per Texas Children’s Hospital Hodgkin disease institutional protocol (TXCH-HD-12A), which utilizes the same chemotherapy backbone as the intermediate-risk Children’s Oncology Group (COG) study, AHOD0031. While 41 subjects were treated per TXCH-HD-12A, 40 were enrolled on the study. Of these, 4 (10%) were slow early responders (SERs) and 36 (90%) were rapid early responders (RERs). Four (10%) relapsed, all within two years of diagnosis (Supplementary Figure S1), and there are no deaths to date (median follow-up: 5 years; range: 2–6 years). These outcomes are similar to the 14% relapse rate, 85% event-free survival (EFS), and 98% overall survival (OS) for AHOD0031 [14].

Fifteen subjects who were not treated with the TXCH-HD-12A regimen were treated per COG protocols. Two subjects with LR/IR nodular lymphocyte predominant disease were treated per AHOD03P1 [28]. All 13 subjects with high-risk (HR) disease were treated per COG HR protocols, AHOD0831 or AHOD1331 [29,30].

### 2.2. The Pre-Therapy Plasma Inflammatory Proteome in Subjects with HL Is Distinct from Controls

**Figure 1:**
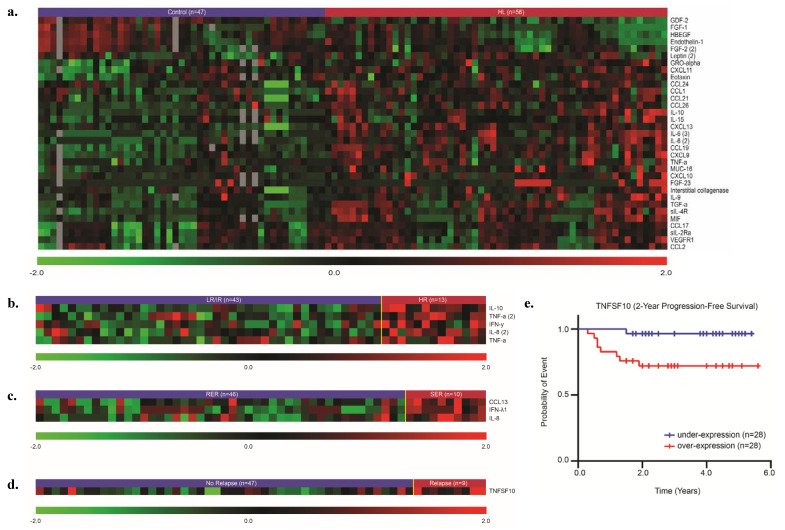
The pre-therapy plasma proteome is distinct from controls and correlates with risk characteristics and outcomes, including therapy response, relapse, and event-free survival. Plasma proteins are compared between the following groups: (**a**) Controls vs. HL, (**b**) LR/IR vs. HR, (**c**) RER vs. SER, (**d**) No Relapse vs. Relapse. (**e**) TNFSF10 is predictive of event-free survival (median expression is used as cut-off). All the significant proteins were controlled at FDR = 0.1.

When comparing the profiles of subjects with HL (*n* = 56) vs. controls (*n* = 47), 32 cytokines or chemokines were significantly different (false discovery rate (FDR) ≤ 0.1) between the two groups (Figure 1a and Supplementary Table S3a). The cytokines/chemokines that were significantly higher in HL included TGF-α (11.11-fold), C-X-C motif chemokine 13 (CXCL13) (6.25-fold), IL-10 (4.35-fold), CXCL9 (4.17-fold), CCL19 (4.00-fold), and CCL17 (3.70-fold). IL-6 was present in multiple Luminex kits (IL-6, IL-6.2, IL-6.3), and thus tested multiple times with distinct analytes. IL-6.2 and IL-6.3 were both significantly elevated (7.14-fold and 5.00-fold, respectively). The cytokines/chemokines that were significantly lower in HL vs. controls included growth differentiation factor 2 (GDF-2; 0.17-fold), heparin-binding EGF-like growth factor (HB-EGF; 0.40-fold), and endothelin-1 (0.54-fold). Twenty of the 32 differentially-expressed analytes had previously been reported in association with HL, and 12 are novel HL-associated analytes (Table S4).

### 2.3. An Inflammatory Signature Distinguishes Subjects with HR HL from Those with LR/IR HL

When comparing subjects with LR/IR disease (*n* = 43) to those with HR disease (*n* = 13), a subset of cytokines/chemokines were significantly elevated among those with HR disease (Figure 1b and Table S3b). These included IL-10 (8.57-fold), IL-8.2 (4.52-fold), IFN-γ (3.01-fold), and TNF-α (tested twice: 2.08-fold and 2.90-fold). IL-10 and TNF receptor have been shown to have prognostic significance in previous studies (Table 1). No analytes were significantly decreased in HR HL.

### 2.4. CCL13, Interferon Lambda-1 (IFN-λ1), and IL-8 Are Elevated in HL Subjects Who Are Slow Early Responders

When comparing RERs (*n* = 46) to SERs (*n* = 10), three cytokines/chemokines were significantly elevated in SERs (Figure 1c and Supplementary Table S3c). These included CCL13 (5.88-fold), IFN-λ1 (5.26-fold), and IL-8 (4.17-fold), none of which have demonstrated prognostic significance previously.

### 2.5. Tumor Necrosis Factor Ligand Superfamily Member 10 (TNFSF10) Is Elevated in Subjects with Relapsed HL and Is Predictive of EFS

When comparing subjects with non-relapsed disease (*n* = 47) to those with relapsed disease (*n* = 9, follow-up: 2–6 years), TNFSF10 was the only cytokine elevated among those with relapsed HL (3.03-fold) (Figure 1d and Table S3d). Similarly, TNFSF10 was also predictive of EFS, or relapse (hazard ratio (HR): 1.999) (Figure 1e). This cytokine has not been previously described in HL.

### 2.6. Cytokines or Chemokines Are Not Significantly Associated with Specific Demographic or Clinical Features

None of the cytokines/chemokines that were significantly different between HL vs. controls, HR vs. LR/IR, SER vs. RER, or relapse vs. no relapse were significantly associated with specific demographic or clinical features (e.g., age (pediatric vs. adolescent/young adult), race/ethnicity, Epstein-Barr virus (EBV) status).

## 3. Discussion

The immune microenvironment that characterizes HL plays an important role in disease pathogenesis; however, its role in pediatric HL has not been well characterized. In this study, we identified 32 unique cytokines/chemokines that distinguish the HL proteomic profile from controls, as well as specific cytokines/chemokines associated with extent of disease (IL-8, IL-10, IFN-γ, TNF-α), therapy response (CCL13, IFN-λ1, and IL-8), and relapse/EFS (TNFSF10). While some of these cytokines/chemokines that play a role in the inflammatory response have been previously reported to be prognostic in adult HL, we found new associations not previously described—IFN-λ1 (therapy response) and TNFSF10 (relapse, EFS).

Prior studies have evaluated the prognostic significance of HL biomarkers, but the objective of the present study was to identify novel biomarkers and validate or invalidate findings of previous studies. Table 1 details pediatric and adult studies that have reported on the cytokines/chemokines evaluated here. Differences between this study and prior pediatric studies may be due to cohort size, inclusion of various cancer diagnoses, or study design (e.g., evaluating the whole lesion rather than serum or plasma; analytes evaluated; statistical methods). These differences may contribute to some differences in this study compared to other HL biomarker investigations. For example, sICAM-1 has been associated with poor outcomes in pediatric HL; this analyte was not included in this study panel. Future studies with focused pediatric HL biomarker candidates will be important to create and validate a reproducible risk-prediction tool.

Results of these prior pediatric studies also do not overlap with observations in adult patients, with the exception of IL-10, which was found to be associated with general symptoms and elevated prior to therapy in non-responders in previous studies [26]. Some cytokine profiling studies in adult studies have reported prognostic significance of several biomarkers. (Reviewed in Diefenbach JNCI 2017 [17]) For example, many have demonstrated the potential importance of CCL17, which is a chemokine expressed by HRS cells in classical HL that contributes to the T-helper type 2 (Th2) cell influx [18,31]. Baseline expression of CCL17 has been associated with Ann Arbor stage and poor outcomes, while changes in CCL17 levels have correlated with treatment response [2,6,18]. While we did not find a significant association between CCL17 and clinical outcomes, CCL17 levels were significantly higher in subjects with HL vs. controls (3.7-fold elevation). Other cytokines/chemokines that play a role in the inflammatory response have been associated with prognosis in adult HL as well, including elevated IL-6, IL-10, TNF receptor, sGal-1, and tumor necrosis factor receptor superfamily member 8 (or sCD30) [22,25,32,33]. Our study demonstrated an elevation of IL-6 in subjects with HL, as well as a prognostic association with both IL-10 and TNF-α (HR disease).

In this study, comprehensive analysis of pre-therapy plasma identified cytokines/chemokines previously associated with adult HL, as well as several novel biomarkers. This approach also identified specific cytokines/chemokines significantly elevated in patients with HR vs. LR/IR disease, including IL-8, IL-10, IFN-γ, and TNF-α. IL-8 is a chemokine produced by macrophages and other cell types [34], and its expression in HL is largely confined to reactive cells and associated with infiltration by neutrophils [35]. While the prognostic significance of macrophages in HL is conflicting [2,25,36,37,38,39], certain proteins secreted by macrophages were associated with HL vs. controls (e.g., growth-regulated alpha protein, TGF-α, and CXCL10) or were related to clinical outcomes (e.g., IL-8) in our study (Table S4). IL-10 is a cytokine that inhibits inflammatory responses by helping to inactivate T cell, monocyte, and macrophage function, and its elevation has been associated with various types of cancer, including HL [40], and outcomes in HL [26]. IL-10 may be produced by the HRS cells, decreasing the natural T cell anti-tumor response, or released by the cells of the surrounding microenvironment in attempt to limit the inflammatory response. IFN-γ is a Th1 cytokine that is responsible for macrophage activation, as well as growth, maturation, and differentiation of various cell types [41]. Both Gerdes et al. and Vassilakopoulos et al. showed that 50–92% of HRS cells in HL biopsy samples were positive for IFN-γ [42,43], suggesting HRS as a possible source of IFN-γ that may contribute to inflammatory microenvironment in HL. Moreover, IFN-γ is known to upregulate programmed death-ligand 1 (PD-L1), which is a mechanism HL uses to evade the immune system. Elevation of IFN-γ was predictive of a positive response to immune checkpoint inhibitors that target PD-L1 [44]; thus, this may be an important biomarker in the immunotherapy of HL. Finally, TNF-α regulates immune cells and plays a role in apoptotic cell death and inflammation [45]. TNF-α elevation has been shown to be associated with worse EFS and OS in univariate but not multivariate analysis [33].

Inflammatory proteins associated with response to therapy, relapse, and EFS are also important to identify, as they may also help to risk stratify patients with HL. Elevated levels of IL-8, IFN-λ1, and CCL13 were significantly associated with slow early response to therapy in this analysis, while TNFSF10 was significantly elevated among subjects who ultimately relapsed and was also predictive of EFS. IFN-λ1 inhibits Th2 responses and upregulates the levels of the inflammatory chemokines IL-6, IL-10, and IL-8 [46], whose role in the reactive cells of HL was described above. Though no previous HL studies have documented the significance of IFN-λ1 in clinical outcomes, this protein may contribute to the slower response to therapy by decreasing anti-tumor activity and increasing inflammation. Similarly, CCL13, which is induced by IL-1 and TNF-α, may contribute to inflammation through its induction of chemotaxis in monocytes, eosinophils, T lymphocytes, and basophils [47]. Expression of CCL13 mRNA has been demonstrated in HRS cells of HL tissues [48].

TNFSF10 has been shown to enhance tumor progression in non-Hodgkin lymphoma by activating NF-κB in apoptosis-resistant cells [49]. The NF-κB pathway plays a role in inflammation, proliferation and apoptosis [31]. In classical HL, this pathway is constitutively activated, promoting HRS cell proliferation and survival through upregulation of proteins that regulate the cell cycle and anti-apoptotic proteins [50]. Elevation of TNFSF10 among relapsed patients may be responsible for re-activation of the NF-κB pathway, leading to relapsed disease.

Few pediatric studies have performed a comprehensive investigation of cytokines/chemokines and their role in outcomes in HL. This study included unbiased analysis of a broad range of proteins associated with immune function in a prospective collection of pre-therapy samples from a previously untreated cohort of pediatric patients with HL. The low proportion of slow early responders (18%) and relapse events (16%), which is expected in this disease group, limited the analysis of these groups. Despite this, significant differences were identified when comparing the proteome within certain subgroups (HR vs. LR/IR; SER vs. RER; Relapse vs. No Relapse). Some of the significant cytokines/chemokines identified when comparing between subgroups were not statistically different comparing all HL vs. controls due to “dilution” of analyte levels from smaller HL subgroups (Supplementary Figure S2). Another feature of this study is that it was from a single institution with a large Hispanic population, so the results may not be generalizable to all populations. However, though a survival difference related to race/ethnicity has been described in pediatric HL [51], none of the significant cytokines/chemokines identified within the subgroups demonstrated a significant association with Hispanic ethnicity. Nonetheless, a next step is to test the candidate clinical biomarkers identified in this analysis in validation cohorts that integrate various populations. The pre-therapy plasma proteome of pediatric subjects with HL in this study was distinct from controls and correlated with some disease characteristics and outcomes. These results indicate potential for pre-therapy plasma biomarkers to be incorporated into biologically-based disease risk stratification to optimize outcomes and minimize toxicities in pediatric HL.

## 4. Materials and Methods

### 4.1. Study Population

The study population included subjects (age < 22 years) diagnosed with childhood HL between 2013 and 2017 at Texas Children’s Hospital. Demographic and clinical data were obtained, including age, sex, ethnicity, HL subtype, risk category (LR/IR: stage IA-B, IIA-B, IIIA, IVA or HR: stage IIIB, IVB), response category (RER or SER), and relapse status (relapse or no relapse) according to the LR/IR TXCH-HD-12A institutional protocol (NCT01858922). This institutional protocol was developed to evaluate the immunological environment within HL in a standardized protocol and utilized response criteria and a treatment strategy based on the COG IR study, AHOD0031 [14]. This study defines RER as having a complete or very good partial response after two cycles of chemotherapy based upon imaging, while SER is defined as having less than a very good partial response. Treatment includes doxorubicin, bleomycin, vincristine, etoposide, prednisone, and cyclophosphamide (ABVE-PC) for all subjects, as well as additional therapy for slow early responders. Most subjects with LR/IR disease received treatment per TXCH-HD-12A. Some subjects with LR/IR nodular lymphocyte predominant HL received treatment according to AHOD03P1, which uses doxorubicin, vincristine, prednisone, and cyclophosphamide (AVPC) as the chemotherapy regimen [28]. Subjects with HR disease were treated according to recent HR COG protocols, AHOD0831 or AHOD1331, in which demographic data and response status were comparably defined [29,30]. Similar to LR/IR subjects, those with HR disease also received ABVE-PC with additional therapy for slow early responders. Controls were pediatric patients who enrolled on a local biology protocol (H-32680) during evaluation for lymphadenopathy but without evidence of malignancy or systemic inflammation (e.g., normal sedimentation rate, C-reactive protein, ferritin). This research was conducted under study protocols approved by the Institutional Review Board for Human Subject Research for Baylor College of Medicine (BCM) and Affiliated Hospitals (ethic code/project identify numbers: H-32680 and H-30993 (NCT01858922) at BCM) and in accordance with the Declaration of Helsinki.

### 4.2. Assessment of Plasma Cytokine/Chemokine Profile

Pre-therapy plasma samples were collected from all evaluable subjects enrolled on local biology (H-32680) and/or treatment (TXCH-HD-12A) protocols. Plasma concentrations of 135 distinct plasma proteins (144 analytes due to some duplications across kits, each treated independently) that have been associated with immune function were measured using the Luminex^®^ platform (Milliplex MAP kits, EMD Millipore) according to the manufacturers’ protocols (Table S5). The associations between cytokine/chemokine concentration and disease characteristics were evaluated.

### 4.3. Statistical Analysis

Plasma protein levels of 135 analytes representing cytokines, chemokines, and growth factors are listed in Table S6. The concentration of each analyte was quantified by comparing results against respective protein standards. A logarithmic (base 2) transformation was applied to the sample concentrations prior to the analysis. The quality control of the samples was performed by detecting outliers using three different metrics: sample–sample correlations (Spearman correlation), >50% missing values per analyte, and overall distribution of log concentrations (box plots). No samples were rejected based on these criteria. The assays were organized to maintain similar proportions of each sample class between plates to minimize batch effects. A univariate *t*-test was used to compare clinical features of HL subjects with healthy controls, LR/IR with HR cases, RER with SER cases, and relapse with non-relapse cases. RER and SER were defined according to AHOD0031 criteria [14]. To control for multiple testing in our dataset, a multivariate permutation test was used to estimate the FDR with a confidence level at 80 percent and the maximum allowed proportion of false-positive proteins at 0.1. The condition ensures that only 10% of significant proteins could be false positives. The permutation test is non-parametric and does not require Gaussian distribution of the data. A survival analysis, also controlled at FDR = 0.1, was performed by fitting a Cox Proportional Hazards model of the concentrations of the analyte with respect to the EFS of the patients. Events were defined by date of relapse, as there were no cases of refractory disease or death in this cohort. Statistical analyses and data processing were performed using BRB-Array Tools version 4.4.0 developed by Dr. Richard Simon and the BRB-Array Tools development Team (http://linus.nci.nih.gov/BRB-ArrayTools.html). Heat maps of the differentially expressed analytes were generated for proteins of interest using R version 3.0.1 (www.r-project.org).

## 5. Conclusions

The pre-therapy plasma cytokine and chemokine profile of pediatric subjects with HL is distinct from controls and correlates with risk characteristics and outcomes (therapy response and EFS). These biomarkers could be incorporated into biologically based risk stratification to optimize outcomes and minimize toxicities in pediatric HL, ultimately helping to improve survival and long-term quality of life for children with HL.

## Figures and Tables

**Table 2:** Demographic and Clinical Features of HL Subjects.

Variation	Value
Sex, *n* (%)	
Male	32 (57)
Female	24 (43)
Age, mean (range)	13 years (3–18 years)
Ethnicity, *n* (%)	
non-Hispanic white	21 (38)
non-Hispanic black	9 (16)
Hispanic	24 (43)
non-Hispanic Asian	2 (3)
HL Subtype, *n* (%)	
Nodular Sclerosing	35 (63)
Mixed Cellularity	12 (21)
Lymphocyte Rich	1 (2)
Classical HL NOS	5 (9)
Nodular Lymphocyte Predominant	3 (5)
EBV positive, *n* (%)	
Yes	18 (32)
No	37 (66)
Unknown	1 (2)
Stage, *n* (%)	
I	4 (7)
II	26 (46)
III	10 (18)
IV	16 (29)
Risk Category, *n* (%)	
LR/IR	43 (77)
HR	13 (23)
HL Therapy Protocol, *n* (%)	
TXCH-HD-12A	41 (73)
AHOD03P1	2 (4)
AHOD0831	12 (21)
AHOD1331	1 (2)
Therapy Response, *n* (%)	
RER	46 (82)
SER	10 (18)
Event-Free Survival, *n* (%)	
Relapse	9 (16)
No Relapse	47 (84)

Abbreviations: HL, Hodgkin lymphoma; NOS, not otherwise specified; EBV, Epstein-Barr virus; LR/IR, low-risk/intermediate-risk; HR, high-risk; TXCH-HD-12A, Texas Children’s Hospital Hodgkin disease institutional protocol; RER, rapid early responder; SER, slow early responder.

## Supplementary Materials

The following are available online at https://www.mdpi.com/2072-6694/12/12/3603/s1, Figure S1: Event-Free Survival for Subjects Enrolled on TXCH-HD-12A (*n* = 40); Figure S2: Significant Cytokines/Chemokines Identified Between Subgroups but Not Between HL vs. Controls; Table S1: Demographic and Clinical Features of Individual HL Subjects; Table S2: Demographic and Clinical Features of Individual Control Subjects; Table S3: Proteins Significant in Study Cohort for (a) HL vs. Controls, (b) HR vs. LR/IR, (c) SER vs. RER, (d) Relapse vs. No Relapse; Table S4: Proteins Significant in Study Cohort; Table S5: Detailed Analyte Information; Table S6: Proteins Analyzed—Luminex vs. Preferred Protein Names.
