# Supplementary material for: Defining the Inflammatory Plasma Proteome in Pediatric Hodgkin Lymphoma
Source: Cancers (Basel). 2020 Dec 2;12(12):3603. doi: 10.3390/cancers12123603 (PMC7761312; doi:10.3390/cancers12123603)
Supplement: Supplementary file 1 [file cancers-12-03603-s001.pdf]

Supplementary Materials:

Defining the Inflammatory Plasma Proteome in Pediatric Hodgkin Lymphoma

Jennifer E. Agrusa, Brooks P. Scull, Harshal A. Abhyankar, Howard Lin, Nmazuo W. Ozuah, Rikhia Chakraborty, Olive S. Eckstein, Nitya Gulati, Elmoataz Abdel Fattah, Nader K. El-Mallawany, Rayne H. Rouce, ZoAnn E. Dreyer, Julienne Brackett, Judith F. Margolin, Joseph Lubega, Terzah M. Horton, Catherine M. Bollard, M. Monica Gramatges, Kala Y. Kamdar, Kenneth L. McClain, Tsz-Kwong Man and Carl E. Allen

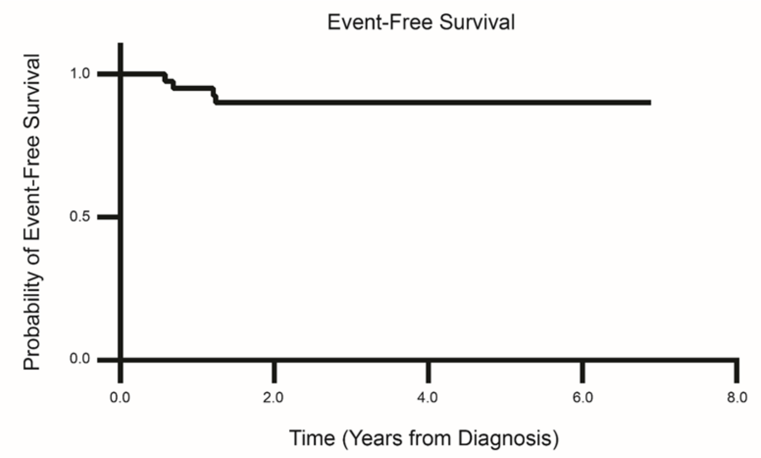

Figure S1. Event-free survival for subjects enrolled on TXCH-HD-12A (n = 40). Four subjects (10%) within this cohort relapsed, all within two years of diagnosis.

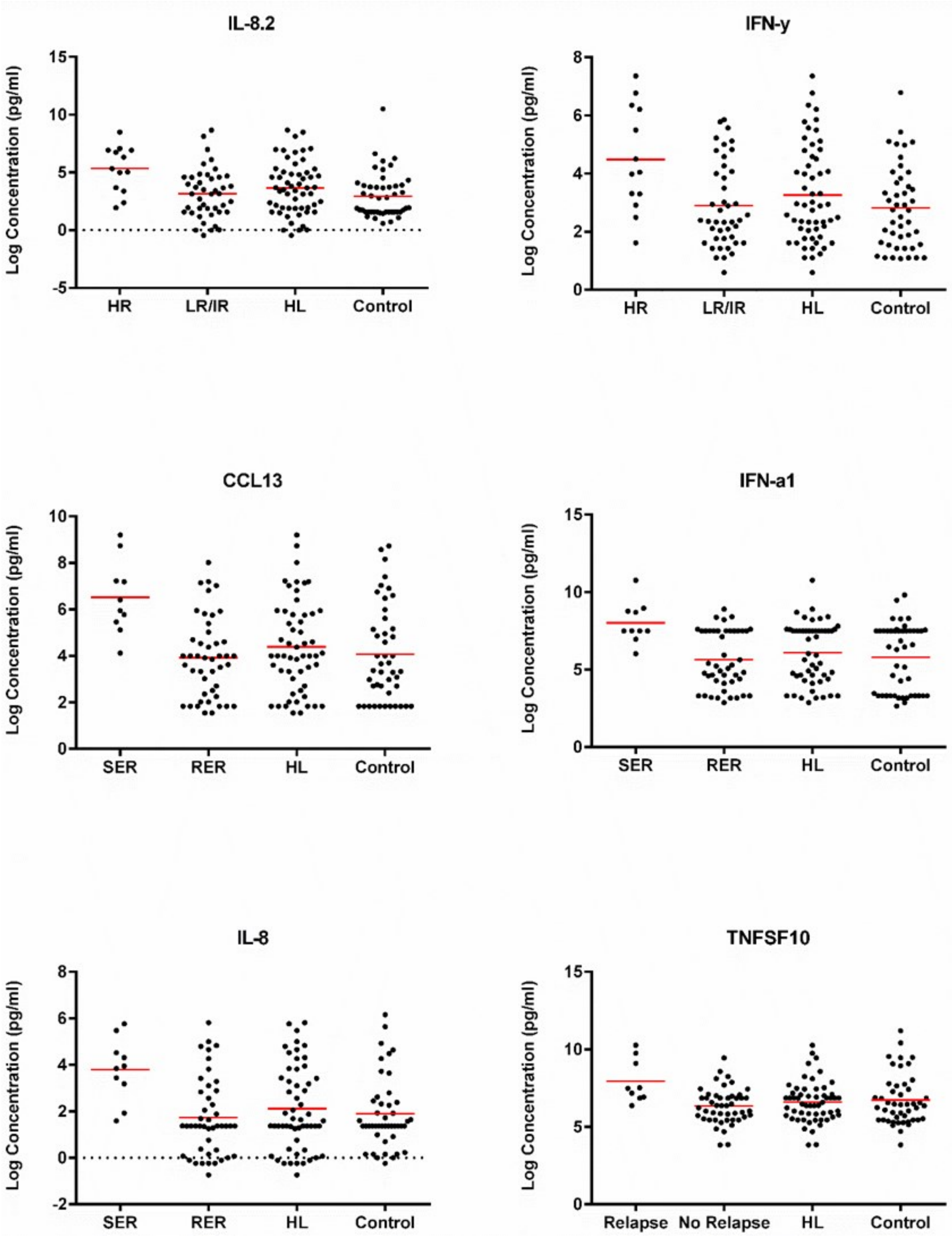

Figure S2. Significant cytokines/chemokines identified when comparing between subgroups were not statistically different comparing all HL vs. controls due to "dilution" of analyte levels from smaller HL subgroups.

Table S1. Demographic and Clinical Features of Individual HL Subjects. F, female; M, male; NOS, not otherwise specified; LP, nodular lymphocyte predominant; LR, lymphocyte-rich; MC, mixed cellularity; NS, nodular sclerosing; LR/IR, low-risk/intermediate-risk; HR, high-risk; RER, rapid early responder; SER, slow early responder.

Subject	Gender	Age	Clinical Diagnosis	Ethnicity	Risk	Response to Therapy	Relapse
**HL01**	F	14	CLASSICAL NOS	Hispanic	HR	RER	No
**HL02**	M	12	CLASSICAL NOS	non-Hispanic black	LR/IR	RER	No
**HL03**	M	11	CLASSICAL NOS	non-Hispanic white	LR/IR	RER	No
**HL04**	M	12	CLASSICAL NOS	Hispanic	LR/IR	SER	No

**HL05**	F	17	CLASSICAL NOS	non-Hispanic white	LR/IR	SER	No
**HL06**	F	8	LP	Hispanic	LR/IR	RER	No
**HL07**	F	13	LP	non-Hispanic white	LR/IR	RER	No
**HL08**	M	11	LP	non-Hispanic black	LR/IR	RER	Yes
**HL09**	M	3	LR	Hispanic	LR/IR	RER	No
**HL10**	M	9	MC	Hispanic	HR	SER	No
**HL11**	M	13	MC	Hispanic	HR	RER	No
**HL12**	F	11	MC	non-Hispanic white	HR	RER	No
**HL13**	M	13	MC	non-Hispanic black	HR	RER	Yes
**HL14**	M	7	MC	non-Hispanic black	LR/IR	RER	No
**HL15**	M	11	MC	Hispanic	LR/IR	RER	No
**HL16**	F	10	MC	Hispanic	LR/IR	RER	No
**HL17**	M	9	MC	non-Hispanic white	LR/IR	RER	No
**HL18**	M	17	MC	non-Hispanic white	LR/IR	RER	No
**HL19**	M	11	MC	non-Hispanic white	LR/IR	RER	No
**HL20**	F	11	MC	non-Hispanic white	LR/IR	RER	No
**HL21**	F	15	MC	non-Hispanic white	LR/IR	RER	Yes
**HL22**	M	14	NS	Hispanic	HR	SER	No
**HL23**	M	17	NS	Hispanic	HR	RER	No
**HL24**	F	13	NS	Hispanic	HR	SER	No
**HL25**	F	17	NS	non-Hispanic white	HR	RER	No
**HL26**	M	12	NS	non-Hispanic white	HR	RER	No
**HL27**	M	12	NS	Hispanic	HR	SER	No
**HL28**	M	15	NS	non-Hispanic Asian	HR	SER	Yes
**HL29**	M	17	NS	non-Hispanic black	HR	SER	Yes
**HL30**	M	5	NS	Hispanic	LR/IR	RER	No
**HL31**	F	6	NS	Hispanic	LR/IR	RER	No
**HL32**	F	13	NS	Hispanic	LR/IR	RER	No
**HL33**	M	9	NS	non-Hispanic white	LR/IR	RER	No
**HL34**	M	17	NS	Hispanic	LR/IR	RER	No
**HL35**	F	12	NS	Hispanic	LR/IR	RER	No
**HL36**	M	18	NS	Hispanic	LR/IR	RER	No
**HL37**	F	15	NS	Hispanic	LR/IR	RER	No
**HL38**	M	12	NS	non-Hispanic white	LR/IR	RER	No
**HL39**	F	7	NS	Hispanic	LR/IR	RER	No
**HL40**	M	16	NS	non-Hispanic white	LR/IR	RER	No
**HL41**	M	16	NS	Hispanic	LR/IR	RER	No
**HL42**	F	17	NS	non-Hispanic white	LR/IR	RER	No
**HL43**	F	17	NS	non-Hispanic black	LR/IR	RER	No
**HL44**	M	7	NS	Hispanic	LR/IR	SER	No
**HL45**	M	11	NS	non-Hispanic white	LR/IR	RER	No
**HL46**	M	16	NS	non-Hispanic white	LR/IR	SER	No
**HL47**	F	16	NS	Hispanic	LR/IR	RER	No
**HL48**	M	16	NS	non-Hispanic white	LR/IR	RER	No
**HL49**	F	16	NS	Hispanic	LR/IR	RER	No
**HL50**	M	13	NS	non-Hispanic black	LR/IR	RER	No
**HL51**	F	16	NS	non-Hispanic white	LR/IR	RER	No
**HL52**	F	13	NS	non-Hispanic black	LR/IR	RER	No
**HL53**	F	14	NS	non-Hispanic white	LR/IR	RER	Yes
**HL54**	M	17	NS	non-Hispanic Asian	LR/IR	RER	Yes
**HL55**	F	17	NS	non-Hispanic white	LR/IR	RER	Yes
**HL56**	F	18	NS	non-Hispanic black	LR/IR	RER	Yes

Table S2. Demographic and Clinical Features of Individual Control Subjects. F, female; M, male; IgM, immunoglobulin

Subject	Gender	Age	Clinical Diagnosis
**C01**	F	16	deep venous thrombosis
**C02**	F	9	anemia
**C03**	F	1	Von Willebrand disease
**C04**	M	2	bruising
**C05**	M	14	anemia
**C06**	F	15	menorrhagia
**C07**	F	17	menorrhagia
**C08**	F	11	vascular malformation
**C09**	F	12	menorrhagia
**C10**	F	5	pyruvate kinase deficiency
**C11**	F	12	menorrhagia
**C12**	M	2	glucose-6-phosphate dehydrogenase (G6PD) deficiency
**C13**	F	16	epilepsy
**C14**	F	1	dermoid cyst, diabetes insipidus
**C15**	F	18	acne
**C16**	M	13	celiac disease
**C17**	F	18	Kikuchi disease
**C18**	F	3	dermoid cyst
**C19**	M	8	rib fracture
**C20**	M	12	ossifying fibroma
**C21**	M	1	dermoid cyst
**C22**	M	18	lymphadenopathy, not lymphoma
**C23**	F	11	non-ossifying fibroma
**C24**	M	13	fibrous dysplasia
**C25**	F	9	vertebra plana (not Langerhans Cell Histiocytosis)
**C26**	M	9	giant cell granuloma
**C27**	F	10	unicentric Castleman disease s/p resection
**C28**	M	6	Gorham-Stout disease
**C29**	F	2	eczema
**C30**	F	6	pituitary stalk tumor (not Langerhans Cell Histiocytosis)
**C31**	F	2	dysuria
**C32**	M	14	collapsed vertebrae
**C33**	M	5	sibling with Langerhans Cell Histiocytosis
**C34**	M	4	vascular malformation
**C35**	M	2	collapsed vertebrae (not Langerhans Cell Histiocytosis)
**C36**	F	1	dermoid cyst
**C37**	M	10	thymic hyperplasia
**C38**	M	17	reactive lymph node
**C39**	M	4	eczema
**C40**	M	5	Bartonella infection
**C41**	M	8	allergies
**C42**	M	15	sibling with genetic disorder
**C43**	M	15	sibling with genetic disorder
**C44**	M	11	desmoid-type fibromatosis
**C45**	M	7	granulomatous lymphadenitis
**C46**	F	19	elevated IgM
**C47**	F	17	reactive lymph node

Table S3. Proteins Significant in Study Cohort for **(a)** HL *vs*. Controls, **(b)** HR *vs*. LR/IR, **(c)** SER *vs*. RER, **(d)** Relapse *vs*. No Relapse.

a.

Analyte	HL Log Average Conc. ± Stdev (pg/mL)	Control Log Average Conc. ± Stdev (pg/mL)	Linear Fold-Change (HL *vs.* Controls)	p-val
**TGF-α**	6.4 ± 3.5	2.9 ± 2.2	11.11	<1E-07
**IL-6.3**	3.4 ± 2.4	0.6 ± 1.1	7.14	<1E-07
**CXCL13**	7.5 ± 1.8	4.9 ± 1.8	6.25	<1E-07
**IL-6.2**	3.4 ± 2.1	1.1 ± 1.4	5	<1E-07
**IL-10**	3.8 ± 2.4	1.7 ± 1.2	4.35	4.00E-07
**CXCL9**	12.0 ± 2.3	9.9 ± 1.5	4.17	8.00E-07
**CCL19**	8.8 ± 1.4	6.9 ± 0.9	4	<1E-07
**CCL17**	10.0 ± 1.4	8.1 ± 3.3	3.7	1.75E-04
**FGF-23**	8.2 ± 4.2	6.5 ± 2.2	3.45	1.32E-02
**CXCL10**	10.1 ± 3.2	8.4 ± 1.4	3.13	2.45E-03
**MIF**	9.7 ± 2.4	8.1 ± 1.1	3.03	5.45E-03
**sIL-4R**	10.7 ± 1.5	9.2 ± 0.9	2.86	<1E-07
**CXCL11**	9.0 ± 1.6	7.6 ± 2.1	2.78	1.07E-04
**IL-9**	3.6 ± 2.4	2.2 ± 1.8	2.63	1.68E-03
**CCL2**	11.9 ± 2.7	10.6 ± 2.6	2.5	1.68E-02
**CCL26**	7.4 ± 1.4	6.3 ± 1.6	2.27	1.33E-04
**GRO-α**	9.6 ± 1.5	8.4 ± 1.9	2.22	8.03E-04
**sIL-2Ra**	12.4 ± 1.2	11.3 ± 1.9	2.17	3.69E-04
**Leptin.2**	12.7 ± 2.2	11.5 ± 2.5	2.17	1.50E-02
**Interstitial Collagenase**	10.5 ± 1.3	9.5 ± 1.7	2.08	6.53E-04
**CCL21**	8.3 ± 0.9	7.3 ± 1.3	1.92	5.06E-05
**MUC-16**	3.0 ± 1.4	2.1 ± 0.8	1.82	3.62E-04
**sVEGFR1**	12.1 ± 1.4	11.3 ± 1.4	1.75	5.06E-03
**CCL24**	7.9 ± 1.4	7.1 ± 1.6	1.72	1.02E-02
**Eotaxin**	7.5 ± 1.3	6.7 ± 1.5	1.67	6.62E-03
**IL-15**	1.7 ± 1.5	0.9 ± 1.3	1.67	1.01-02
**CCL1**	2.3 ± 0.8	1.7 ± 0.8	1.59	3.32E-05
**TNF-α**	3.3 ± 1.1	2.8 ± 0.8	1.41	1.23E-02
**FGF-2.2**	6.8 ± 1.2	7.5 ± 1.1	0.64	7.20E-03
**FGF-1**	4.5 ± 1.0	5.2 ± 1.2	0.6	1.03E-03
**Endothelin-1**	4.2 ± 1.4	5.1 ± 1.4	0.54	1.63E-03
**HB-EGF**	3.7 ± 1.5	5.0 ± 1.6	0.4	5.10E-05
**GDF-2**	4.8 ± 1.9	7.4 ± 1.5	0.17	<1E-07

b.

Analyte	HR Log Average Conc. ± Stdev (pg/mL)	LR/IR Log Average Conc. ± Stdev (pg/mL)	Linear Fold-Change (HR *vs.* LR/IR)	p-val
**IL-10**	6.2 ± 2.2	3.1 ± 1.9	8.57	9.00E-06
**IL-8.2**	5.3 ± 2.0	3.1 ± 2.1	4.52	1.93E-03
**IFN-ɤ**	4.5 ± 1.8	2.9 ± 1.5	3.01	1.89E-03
**TNF-α.2**	3.8 ± 0.9	2.3 ± 1.3	2.9	3.42E-04
**TNF-α**	4.1 ± 1.2	3.0 ± 1.0	2.08	2.34E-03

c.

Analyte	RER Log Average Conc. ± Stdev (pg/mL)	SER Log Average Conc. ± Stdev (pg/mL)	Linear Fold-Change (SER *vs.* RER)	p-val
**CCL13**	3.9 ± 1.7	6.5 ± 1.6	5.88	5.18E-05
**IFN-λ1**	5.6 ± 1.9	8.0 ± 1.3	5.26	2.84E-04
**IL-8**	1.7 ± 1.6	3.8 ± 1.3	4.17	5.45E-04

d.

Analyte	No Relapse Log Average Conc. ± Stdev (pg/mL)	Relapse Log Average Conc. ± Stdev (pg/mL)	Linear Fold-Change (Relapse vs. No Relapse)	p-val
**TNFSF10**	6.3 ± 1.1	7.9 ± 1.4	3.03	4.84E-04

Each comparison is significant at FDR=0.1.

Abbreviations: HL, Hodgkin lymphoma; Conc; concentration; Stdev; standard deviation; HR, high-risk; LR/IR, low-risk/intermediate-risk; SER, slow early responder; RER, rapid early responder; pg/mL, picograms per milliliter.

Table S4. Proteins Significant in Study Cohort.

Protein	Alternative Protein Name(s)	Tissue and/or Cellular Source of Protein	Brief Function	Previously reported in HL
HL vs. Controls				
C-C motif chemokine 1 (CCL1)	Small-inducible cytokine A1<br>T lymphocyte-secreted protein I-309	Widely expressed, reported in lung; dendritic cells, dermal endothelial cells	Attracts monocytes, NK cells, and immature B cells and dendritic cells	Yes (Hanamoto et al., 2004)
C-C motif chemokine 2 (CCL2)	HC11<br>Monocyte chemoattractant protein 1<br>Monocyte chemotactic and activating factor (MCAF)<br>Monocyte chemotactic protein 1 (MCP-1)<br>Monocyte secretory protein<br>Small-inducible cytokine A2	Widely expressed, reported in smooth muscle; monocytes	Chemotactic for monocytes and basophils	Yes (Teruya-Feldstein, Tosato, & Jaffe, 2000)
C-C motif chemokine 17 (CCL17)	CC chemokine TARC<br>Small-inducible cytokine A17<br>Thymus and activation-regulated chemokine (TARC)	Widely expressed, reported in lymph node; cells of thymus, peripheral blood mononuclear cells	Chemotactic activity for T cells and other leukocytes	Yes<br>(Jones et al., 2013; Niens et al., 2008)
C-C motif chemokine 21 (CCL21)	6CKine<br>Beta-chemokine exodus-2<br>Secondary lymphoid-tissue chemokine (SLC)<br>Small-inducible cytokine A21	Widely expressed, reported in adrenal gland; endothelial cells, dendritic cells	Chemotactic *in vitro* for thymocytes and activated T cells; mediates homing of lymphocytes to secondary lymphoid organs	Yes (Machado et al., 2009)
C-X-C motif chemokine 9 (CXCL9)	Gamma-interferon-induced monokine<br>Monokine induced by interferon-gamma<br>(HuMIG, MIG)<br>Small-inducible cytokine B9	Widely expressed, reported in lung; endothelial cells, fibroblasts, neutrophils, keratinocytes	T-cell chemoattractant, induced by IFN-γ; Th1 recruitment	Yes (Skinnider & Mak, 2002)
C-X-C motif chemokine 10 (CXCL10)	10 kDa interferon gamma-induced protein<br>(Gamma-IP10, IP-10)<br>Small-inducible cytokine B10	Widely expressed, reported in ectocervix; monocytes, neutrophils, endothelial cells, keratinocytes, fibroblasts, mesenchymal cells, dendritic cells, hepatocytes, astrocytes	Chemoattractant for monocytes/macrophages, T cells, NK cells, and dendritic cells; promotes T cell adhesion to endothelial cells; antitumor activity; inhibits bone marrow colony formation and angiogenesis	Yes (Teichmann, Meyer, Beck, & Niedobitek, 2005)
C-X-C motif chemokine 11 (CXCL11)	Beta-R1<br>H174<br>Interferon gamma-inducible protein 9 (IP-9)<br>Interferon-inducible T-cell alpha chemoattractant<br>(I-TAC)<br>Small-inducible cytokine B11	Bronchus; endothelial cells, monocytes, epithelial cells, neutrophils, keratinocytes	Induces chemotactic response in activated T cells; acts as chemoattractant for IL-2 activated T cells	Yes (Chetaille et al., 2009)

C-X-C motif chemokine 13 (CXCL13)	Angie<br>B cell-attracting chemokine 1 (BCA-1)<br>B lymphocyte chemoattractant<br>CXC chemokine BLC<br>Small-inducible cytokine B13	Widely expressed, reported in tonsil; marginal reticular cells, follicular dendritic cells, follicular stromal cells	Organizes B cell follicles and germinal centers; promotes migration of B cells	Yes (Nam-Cha et al., 2009)
Endothelin-1	Preproendothelin-1 (PPET1)	Widely expressed, reported in adrenal gland; endothelial cells	Vasoconstriction	Yes (Banerjee, 2011)
Eotaxin	C-C motif chemokine 11<br>Eosinophil chemotactic protein<br>Small-inducible cytokine A11	Widely expressed, reported in stomach; activated monocytes, T lymphocytes	Recruits eosinophils	Yes (Salcedo et al., 2001)
Fibroblast growth factor 1 (FGF-1)	Acidic fibroblast growth factor (aFGF)<br>Endothelial cell growth factor (ECGF)<br>Heparin-binding growth factor 1 (HBGF-1)	Kidney, brain	Modifies endothelial cell migration and proliferation; angiogenic	Yes (Khnykin, Troen, Berner, & Delabie, 2006)
Fibroblast growth factor 2 (FGF-2)	Basic fibroblast growth factor (bFGF)<br>Heparin-binding growth factor 2 (HBGF-2)	Widely expressed, reported in cartilage; epithelial cells	Regulates cell survival, cell division, cell differentiation, cell migration; induces angiogenesis	Yes (Gharbaran et al., 2013; Khnykin et al., 2006)
Growth-regulated alpha protein (GRO-α)	C-X-C motif chemokine 1<br>GRO-alpha(1-73)<br>Melanoma growth stimulatory activity (MGSA)<br>Neutrophil-activating protein 3 (NAP-3)	Widely expressed, reported in smooth muscle; macrophages, neutrophils, epithelial cells	Neutrophil chemoattractant	Yes (Skinnider & Mak, 2002)
Interleukin-6 (IL-6)	B-cell stimulatory factor 2 (BSF-2)<br>CTL differentiation factor (CDF)<br>Hybridoma growth factor<br>Interferon beta-2 (IFN-ß-2)	Widely expressed, reported in left coronary artery; macrophages, Th2 cells, B cells, astrocytes, endothelial cells	B cell maturation, mediates acute phase response, stimulates IL-1 and TNF-α production	Yes (Reynolds et al., 2002)
Interleukin-9 (IL-9)	Cytokine P40<br>T-cell growth factor P40	Widely expressed, reported in tibial nerve; Th2 cells	Regulates variety of hematopoietic cells; stimulates cell proliferation and prevents apoptosis	Yes (Rojas-Zuleta & Sanchez, 2017)
Interleukin-10 (IL-10)	Cytokine synthesis inhibitory factor (CSIF)	Widely expressed, reported in esophagus; T helper cells, monocytes, macrophages and dendritic cells, various immune effector cells	Immunosuppressive; growth and differentiation factor for B cells	Yes (Skinnider & Mak, 2002)
Interleukin-15 (IL-15)	None	Decidua; dendritic cells, keratinocytes, fibroblasts, myocyte, nerve cells	Stimulates proliferation of T cells	Yes (Ullrich et al., 2015)

Interstitial collagenase	Fibroblast collagenase<br>Matrix metalloproteinase-1 (MMP-1)	Widely expressed, reported in smooth muscle; macrophages, smooth muscle, endothelial cells, fibroblasts	Breaks down interstitial collagens	Yes (Kasim 2014)
Macrophage migration inhibitory factor (MIF)	Glycosylation-inhibiting factor (GIF)<br>L-dopachrome isomerase<br>L-dopachrome tautomerase<br>Phenylpyruvate tautomerase	Widely expressed, reported in adrenal gland; macrophages, lymphocytes	Pro-inflammatory; mediates macrophage function	Yes (Renner & Stenner, 2018)
Tumor necrosis factor (TNF)	Cachectin<br>TNF-alpha, TNF ligand superfamily member 2 (TNFα)	Leukocytes	Immunoregulatio n	Yes (Hoppe, 1999)
C-C motif chemokine 19 (CCL19)	Beta-chemokine exodus-3<br>CK beta-11<br>Epstein-Barr virus-induced molecule 1 ligand chemokine (ELC)<br>Macrophage inflammatory protein 3 beta (MIP-3β)<br>Small-inducible cytokine A19	Widely expressed, reported in appendix; endothelial cells, T cell zones in lymph nodes, dendritic cells	Lymphocyte recirculation and homing, trafficking T cells in thymus, chemotactic activity for T/B cells	No
C-C motif chemokine 24 (CCL24)	CK-beta-6<br>Eosinophil chemotactic protein 2<br>Eotaxin-2<br>Myeloid progenitor inhibitory factor 2 (MPIF-2)<br>Small-inducible cytokine A24	Widely expressed, reported in spleen; activated monocytes, T lymphocytes	Induces chemotaxis in eosinophils; strongly chemotactic for resting T cells, slightly chemotactic for neutrophils	No
C-C motif chemokine 26 (CCL26)	CC chemokine IMAC<br>Eotaxin-3<br>Macrophage inflammatory protein 4-α (MIP-4-α)<br>Small-inducible cytokine A26<br>Thymic stroma chemokine-1 (TSC-1)	Widely expressed, reported in adenohypophysis; endothelial cells, dermal fibroblasts	Chemotactic for eosinophils and basophils	No
Fibroblast growth factor 23 (FGF-23)	Phosphatonin<br>Tumor-derived hypophosphatemia-inducing factor	Widely expressed, reported in heart; osteoblasts	Regulates phosphate homeostasis; negatively regulates osteoblast differentiation and matrix mineralization	No
Growth differentiation factor 2 (GDF-2)	bone morphogenetic protein 9 (BMP-9)	Widely expressed, reported in liver; hepatocytes	Inhibits proliferation and induces apoptosis in many cell types, including B cells	No
Heparin-binding EGF-like growth factor (HB-EGF)	None	Widely expressed, reported in synovial joint; monocytes/macro phages, CD4+ lymphocytes, neutrophils, eosinophils,	Influences cell cycle progression, molecular chaperone regulation, cell survival, cellular functions, adhesion, and	No

		endothelial cells, epithelial cells	mediation of cell migration	
Leptin	Obese protein<br>Obesity factor	Adipose tissue; adipocytes	Pro-angiogenic, pro-inflammatory and mitogenic factor; crosstalks with IL-1 family cytokines in cancer	No
Mucin-16 (MUC-16)	Ovarian cancer-related tumor marker CA125 (CA125)<br>Ovarian carcinoma antigen CA125	Widely expressed, reported in nasal cavity; epithelial cells	Suppresses response of natural killer cells; binds galectin-1 (immunosuppressive protein)	No
Soluble interleukin-2 receptor alpha (sIL2Rα)	TAC antigen<br>p55<br>CD_antigen: CD25	Widely expressed, reported in lymph node; peripheral blood mononuclear cells	Antagonist of IL-2 mediated responses; marker of T cell activation	No
Soluble interleukin-4 receptor alpha (sIL-4Rα)	CD_antigen: CD124	Widely expressed, reported in adrenal gland; lymphocytes	Immunoregulation	No
Soluble vascular endothelial growth factor receptor 1 (sVEGFR-1)	Fms-like tyrosine kinase 1 (FLT-1)<br>Tyrosine-protein kinase FRT<br>Tyrosine-protein kinase receptor FLT (FLT)<br>Vascular permeability factor receptor	Widely expressed, reported in placenta; endothelial cells, monocytes.	Inhibitor of angiogenic signaling	No
Transforming growth factor alpha (TGFα)	EGF-like TGF (ETGF)<br>TGF type 1	Widely expressed, reported in retina; macrophages	Induces epithelial development and cell proliferation; tumorigenesis; angiogenesis	No
Risk (LR/IR *vs.* HR)				
Interferon gamma (IFN-γ)	Immune interferon	Leukocytes	Activates macrophages, induces Class II major histocompatibility complex molecule expression; CD4+ cell differentiation	Yes (Fozza & Longinotti, 2011; Gerdes et al., 1990)
Interleukin-8 (IL-8)	C-X-C motif chemokine 8<br>C-X-C motif ligand 8<br>Emoctakin<br>Granulocyte chemotactic protein 1 (GCP-1)<br>Monocyte-derived neutrophil chemotactic factor (MDNCF)<br>Monocyte-derived neutrophil-activating peptide (MONAP)<br>Neutrophil-activating protein 1 (NAP-1)<br>Protein 3-10C<br>T-cell chemotactic factor	Widely expressed, reported in periodontal ligament; macrophages, epithelial cells, smooth muscle cells, endothelial cells	Induces granulocyte chemotaxis; promotes angiogenesis	Yes (Foss et al., 1996; Luciani, Stoppacciaro, Peri, Mantovani, & Ruco, 1998)
Interleukin-10 (IL-10)	As above	As above	As above	As above

Tumor necrosis factor (TNF)	As above	As above	As above	As above
Response (RER *vs.* SER)				
C-C motif chemokine 13 (CCL13)	CK-beta-10<br>Monocyte chemoattractant protein 4<br>Monocyte chemotactic protein 4 (MCP-4)<br>NCC-1<br>Small-inducible cytokine A13	Widely expressed, reported in small intestine; chondrocytes, epithelial cells	Induces chemotaxis in monocytes, eosinophils, T cells and basophils	Yes (Maggio et al., 2002; Teruya-Feldstein et al., 2000)
Interleukin-8 (IL-8)	As above	As above	As above	As above
Interferon lambda-1 (IFN-λ1)	Cytokine Zcyto21<br>Interleukin-29 (IL-29)	Leukocytes	Inhibits Th2 responses; upregulates inflammatory chemokines	No
Relapse (Relapse *vs.* No Relapse)				
Tumor necrosis factor ligand superfamily member 10 (TNFSF10)	Apo-2 ligand (Apo-2L)<br>TNF-related apoptosis-inducing ligand (Protein TRAIL)<br>CD_antigen: CD253	Widely expressed, reported in bronchus; epithelial cells	Induces apoptosis	No

Table S4 Bibliography

1. Banerjee, D. (2011). Recent Advances in the Pathobiology of Hodgkin's Lymphoma: Potential Impact on Diagnostic, Predictive, and Therapeutic Strategies. Advances in Hematology, 2011, 439456. doi: 10.1155/2011/439456
2. Chetaille, B., Bertucci, F., Finetti, P., Esterni, B., Stamatoullas, A., Picquenot, J. M., . . . Xerri, L. (2009). Molecular profiling of classical Hodgkin lymphoma tissues uncovers variations in the tumor microenvironment and correlations with EBV infection and outcome. Blood, 113(12), 2765-3775. doi: 10.1182/blood-2008-07-168096
3. Foss, H. D., Herbst, H., Gottstein, S., Demel, G., Araujo, I., & Stein, H. (1996). Interleukin-8 in Hodgkin's disease. Preferential expression by reactive cells and association with neutrophil density. American Journal of Pathology, 148(4), 1229-1236.
4. Fozza, C., & Longinotti, M. (2011). T-Cell Traffic Jam in Hodgkin's Lymphoma: Pathogenetic and Therapeutic Implications. Advances in Hematology, 2011, 501659. doi: 10.1155/2011/501659
5. Gerdes, J., Kretschmer, C., Zahn, G., Ernst, M., Jones, D. B., & Flad, H. D. (1990). Immunoenzymatic assessment of interferon-gamma in Hodgkin and Sternberg-Reed cells. Cytokine, 2(4), 307-310.
6. Gharbaran, R., Goy, A., Tanaka, T., Park, J., Kim, C., Hasan, N., . . . Suh, K. S. (2013). Fibroblast growth factor-2 (FGF2) and syndecan-1 (SDC1) are potential biomarkers for putative circulating CD15+/CD30+ cells in poor outcome Hodgkin lymphoma patients. Journal of Hematology & Oncology, 6, 62. doi: 10.1186/1756-8722-6-62
7. Hanamoto, H., Nakayama, T., Miyazato, H., Takegawa, S., Hieshima, K., Tatsumi, Y., . . . Yoshie, O. (2004). Expression of CCL28 by Reed-Sternberg cells defines a major subtype of classical Hodgkin's disease with frequent infiltration of eosinophils and/or plasma cells. American Journal of Pathology, 164(3), 997-1006. doi: 10.1016/s0002-9440(10)63187-2
8. Hoppe, R. T., et al. (1999). Cytokines, Cytokine Receptors, and Chemokines in Hodgkin Lymphoma. In R. T. Hoppe, et al. (Ed.), Hodgkin Lymphoma 2nd Edition (pp. 93). Philadelphia, PA: Lippincott Williams & Wilkins.
9. Jones, K., Vari, F., Keane, C., Crooks, P., Nourse, J. P., Seymour, L. A., . . . Gandhi, M. K. (2013). Serum CD163 and TARC as disease response biomarkers in classical Hodgkin lymphoma. Clinical Cancer Research, 19(3), 731-742. doi: 10.1158/1078-0432.ccr-12-2693

10. Kasim, Z. M. a. Y., Wasan H. (2014). Immunohistochemical Expression of MMP1 and TIMP1 as Markers of Migration in Hodgkin's and Non - Hodgkin's Lymphoma of the Head and Neck Region: A Comparative Study. College of Dentistry: Baghdad University, 26(3), 72-78.
11. Khnykin, D., Troen, G., Berner, J. M., & Delabie, J. (2006). The expression of fibroblast growth factors and their receptors in Hodgkin's lymphoma. Journal of Pathology, 208(3), 431-438. doi: 10.1002/path.1900
12. Luciani, M. G., Stoppacciaro, A., Peri, G., Mantovani, A., & Ruco, L. P. (1998). The monocyte chemotactic protein a (MCP-1) and interleukin 8 (IL-8) in Hodgkin's disease and in solid tumours. Molecular Pathology, 51(5), 273-276. doi: 10.1136/mp.51.5.273
13. Machado, L., Jarrett, R., Morgan, S., Murray, P., Hunter, B., Hamilton, E., . . . Lee, S. P. (2009). Expression and function of T cell homing molecules in Hodgkin's lymphoma. Cancer Immunology, Immunotherapy, 58(1), 85-94. doi: 10.1007/s00262-008-0528-z
14. Maggio, E., van den Berg, A., Diepstra, A., Kluiver, J., Visser, L., & Poppema, S. (2002). Chemokines, cytokines and their receptors in Hodgkin's lymphoma cell lines and tissues. Annals of Oncology, 13 Suppl 1, 52-56. doi: 10.1093/annonc/13.s1.52
15. Nam-Cha, S. H., Montes-Moreno, S., Salcedo, M. T., Sanjuan, J., Garcia, J. F., & Piris, M. A. (2009). Lymphocyte-rich classical Hodgkin's lymphoma: distinctive tumor and microenvironment markers. Modern Pathology, 22(8), 1006-1015. doi: 10.1038/modpathol.2009.54
16. Niens, M., Visser, L., Nolte, I. M., van der Steege, G., Diepstra, A., Cordano, P., . . . van den Berg, A. (2008). Serum chemokine levels in Hodgkin lymphoma patients: highly increased levels of CCL17 and CCL22. British Journal of Haematology, 140(5), 527-536. doi: 10.1111/j.1365-2141.2007.06964.x
17. Renner, C., & Stenner, F. (2018). Cancer Immunotherapy and the Immune Response in Hodgkin Lymphoma. Frontiers in Oncology, 8, 193. doi: 10.3389/fonc.2018.00193
18. Reynolds, G. M., Billingham, L. J., Gray, L. J., Flavell, J. R., Najafipour, S., Crocker, J., Murray, P. G. (2002). Interleukin 6 expression by Hodgkin/Reed-Sternberg cells is associated with the presence of 'B' symptoms and failure to achieve complete remission in patients with advanced Hodgkin's disease. British Journal of Haematology, 118(1), 195-201.
19. Rojas-Zuleta, W. G., & Sanchez, E. (2017). IL-9: Function, Sources, and Detection. Methods in Molecular Biology, 1585, 21-35. doi: 10.1007/978-1-4939-6877-0_2
20. Salcedo, R., Young, H. A., Ponce, M. L., Ward, J. M., Kleinman, H. K., Murphy, W. J., & Oppenheim, J. J. (2001). Eotaxin (CCL11) induces in vivo angiogenic responses by human CCR3+ endothelial cells. Journal of Immunology, 166(12), 7571-7578. doi: 10.4049/jimmunol.166.12.7571
21. Skinnider, B. F., & Mak, T. W. (2002). The role of cytokines in classical Hodgkin lymphoma. Blood, 99(12), 4283-4297. doi: 10.1182/blood-2002-01-0099
22. Teichmann, M., Meyer, B., Beck, A., & Niedobitek, G. (2005). Expression of the interferon-inducible chemokine IP-10 (CXCL10), a chemokine with proposed anti-neoplastic functions, in Hodgkin lymphoma and nasopharyngeal carcinoma. Journal of Pathology, 206(1), 68-75. doi: 10.1002/path.1745
23. Teruya-Feldstein, J., Tosato, G., & Jaffe, E. S. (2000). The role of chemokines in Hodgkin's disease. Leukemia and Lymphoma, 38(3-4), 363-371. doi: 10.3109/10428190009087027
24. Ullrich, K., Blumenthal-Barby, F., Lamprecht, B., Kochert, K., Lenze, D., Hummel, M., . . . Janz, M. (2015). The IL-15 cytokine system provides growth and survival signals in Hodgkin lymphoma and enhances the inflammatory phenotype of HRS cells. Leukemia, 29(5), 1213-1218. doi: 10.1038/leu.2014.345

Table S5. Detailed Analyte Information.

Millipore Kit	Catalog Number	Dilution Factor	Analytes
**Human Cytokine/Chemokine Panel I**	HCYTMAG-60K-PX38	Neat	EGF, FGF2, Eotaxin, TGF-α, G-CSF, Flt-3L, GM-CSF, Fractalkine, IFN-α2, IFN-γ, GRO, IL-10, MCP-3, IL-12p40, MDC, IL12p70, IL-13, IL-15, sCD40L, IL-17a, IL-1RA, IL-1α, IL-9, IL-1B, IL-2, IL-3, IL-4, IL-5, IL-6, IL-7, IL-8, IP-10, MCP-1, MIP-1α, MIP-1ß, TNF-α, TNF-ß, VEGF
**Human Cytokine/Chemokine Panel II**	HCP2MAG-52K-PX23	Neat	Eotaxin-2, MCP-2, BCA-1, MCP-4, I-309, IL-16, TARC, 6CKine, Eotaxin-3, LIF, TPO, SCF, TSLP, IL-33, IL-20, IL-21, TRAIL, CTACK, SDF-1-α+β, ENA-78, MIP-1δ, IL-28a, IL-23
**Human Cytokine/Chemokine Panel III**	HCYP3MAG-63K	Neat	CXCL6/GCP-2, CXCL11/I-TAC, CCL19/MIP-3ß, CCL20/MIP-3α, XCL1/Lymphotactin, IL-11, IL-29/IFN-λ1, CXCL9/MIG, M-CSF
**RANTES**	HCYTOMAG-60K-01	1:100	RANTES
**Human Circulating Cancer Biomarker Panel 1**	HCCBP1MAG-58K	1:6	AFP, Total PSA, CA15-3, CA19-9, MIF, TRAIL, LEPTIN, IL-6, sFASl, CEA, CA125, IL-8, HGF, sFAS, TNFα, Prolactin, SCF, CYFRA21-1, OPN, FGF2, ß-HCG, HE4, TGFα, VEGF
**Human Circulating Cancer Biomarker Panel 2**	HCCBP2MAG-58K-05	1:10,000	Vitronectin, ECM1, Vitamin D BP, Antithrombin III, CFH
**Human Angiogenesis**	HAGP1MAG-12K-17	1:3	EGF, Angiopoietin-2, G-CSF, BMP-9, Endogolin, Endothelin-1, Leptin, FGF-1, Follistatin, IL-8, HGF, HB-EGF, PLGF, VEGF-C, VEGF-D, FGF-2, VEGF-A
**Human Bone Panel**	HBNMAG-51K-13	1:2	ACTH, DKK1, FGF-23, Insulin, Leptin, OPG, OC, OPN, PTH, SOST, IL-1ß, PTH, FGF-23
**TGF-B**	TGFBMAG-64K-03	1:30	TGFB1, TGFB2, TGFB3
**Human Sepsis Panel 2**	HSP2MAG-63K-07	Neat	Granzyme B, HSP70, MMP-8, IL-8, MIP-1α, MIP-1ß, MMP-8
**MMP Panel 2**	HMMP2MAG-55K-05	1:20	MMP-1, MMP-2, MMP-7, MMP-9, MMP-10
**MMP Panel 1**	HMMP1MAG-55K-03	Neat	MMP-3, MMP-12, MMP-13
**Human TIMP Panel 1**	HTMP1MAG-54K-02	1:50	TIMP-1, TIMP-2
**Human Soluble Cytokine Receptor**	HSCRMAG32KPX14	1:5	sCD30, sEGFR, sgp130, sIL-1RI, sIL-1RII, sIL-2Ra, sIL-4R, sIL-6R, sRAGE, sTNFRI, sTNFrII, sVEGFR1, sVEGFR2, sVEGFR3

Table S6. Proteins Analyzed - Luminex *vs.* Preferred.

**Luminex Name or Abbreviation**	**Preferred Protein Name (Abbreviation)**	**Luminex Name or Abbreviation**	**Preferred Protein Name (Abbreviation)**	**Luminex Name or Abbreviation**	**Preferred Protein Name (Abbreviation)**
6CKine	C-C motif chemokine 21 (CCL21)	DKK1	Dickkopf-related protein 1 (DKK1)	HE4	WAP four-disulfide core domain protein 2
ACTH	Pro-opiomelanocortin (POMC)	ECM1	Extracellular matrix protein 1 (ECM1)	HGF	Hepatocyte Growth Factor
AFP	Alpha-fetoprotein (AFP)	EGF	Pro-epidermal growth factor (EGF)	HSP70	Heat shock 70 kDa protein 1A
Angiopoietin-2	Angiopoietin-2 (ANG-2)	ENA-78	C-X-C motif chemokine 5 (CXCL5)	I-309	C-C Motif Chemokine Ligand 1 (CCL1)
Antithrombin III	Antithrombin-III (ATIII)	Endoglin	Endoglin	IFN-α2	Interferon alpha-2 (IFN-α2)
BCA-1	C-X-C motif chemokine 13 (CXCL13)	Endothelin-1	Endothelin-1	IFN-ɣ	Interferon gamma (IFN-ɣ)
β-HCG	Choriogonadotropin subunit beta 3	Eotaxin	Eotaxin	IL-1α	Interleukin-1 alpha (IL-1α)
BMP-9	Growth/differentiation factor 2 (GDF-2)	Eotaxin-2	C-C motif chemokine 24 (CCL24)	IL-1β	Interleukin-1 beta (IL-1β)
CA125	Mucin-16 (MUC-16)	Eotaxin-3	C-C motif chemokine 26 (CCL26)	IL-1RA	Interleukin-1 receptor antagonist protein (IL-1RA)
CA15-3	Mucin-1 (MUC-1)	FGF-1	Fibroblast growth factor 1 (FGF-1)	IL-2	Interleukin-2 (IL-2)
CA19-9	Alpha-N-acetylgalactosa-minide alpha-2,6-sialyltransferase 6	FGF-2	Fibroblast growth factor 2 (FGF-2)	IL-3	Interleukin-3 (IL-3)
CCL19/MIP-3β	C-C motif chemokine 19 (CCL19)	FGF-23	Fibroblast growth factor 23 (FGF-23)	IL-4	Interleukin-4 (IL-4)
CCL20/MIP-3α	C-C motif chemokine 20 (CCL20)	Flt-3L	FMS-related tyrosine kinase 3 ligand (Flt-3L)	IL-5	Interleukin-5 (IL-5)
CEA	Carcinoembryonic antigen	Follistatin	Follistatin (FS)	IL-6	Interleukin-6 (IL-6)
CFH	Complement factor H (CFH)	Fractalkine	Fractalkine	IL-7	Interleukin-7 (IL-7)
CTACK	C-C motif chemokine 27 (CCL27)	G-CSF	Granulocyte colony-stimulating factor (G-CSF)	IL-8	Interleukin-8 (IL-8)
CXCL11/I-TAC	C-X-C motif chemokine 11 (CXCL11)	GM-CSF	Granulocyte-macrophage colony-stimulating factor (GM-CSF)	IL-9	Interleukin-9 (IL-9)
CXCL6/GCP-2	C-X-C motif chemokine 6 (CXCL6)	Granzyme-B	Granzyme B	IL-10	Interleukin-10 (IL-10)
CXCL9/MIG	C-X-C motif chemokine 9 (CXCL9)	GRO	Growth-regulated alpha protein (GRO-α)	IL-11	Interleukin-11 (IL-11)
CYFRA21-1	CYFRA21-1	HB-EGF	Proheparin-binding EGF-like growth factor (HBEGF)	IL-12(p40)	Interleukin-12 subunit beta (IL-12B)

IL-12(p70)	Interleukin-12 subunit p70 (IL-12[p70])	MCP-4	C-C motif chemokine 13 (CCL13)	OPN	Osteopontin
IL-13	Interleukin-13 (IL-13)	M-CSF	Macrophage colony-stimulating factor 1 (M-CSF)	PLGF	Placenta growth factor (PlGF)
IL-15	Interleukin-15 (IL-15)	MDC	C-C motif chemokine 22 (CCL22)	Prolactin	Prolactin (PRL)
IL-16	Pro-interleukin-16 (IL-16)	MIF	Macrophage migration Inhibitory factor (MIF)	PTH	Prolactin (PRL)
IL-17A	Interleukin-17A (IL-17A)	MIP-1α	C-C motif chemokine 3 (CCL3)	RANTES	Parathyroid hormone (PTH)
IL-20	Interleukin-20 (IL-20)	MIP-1β	C-C motif chemokine 4 (CCL4)	sCD30	C-C motif chemokine 5
IL-21	Interleukin-21 (IL-21)	MIP-1δ	C-C motif chemokine 15 (CCL15)	SCD-40L	Tumor necrosis factor receptor superfamily member 8
IL-23	Interleukin-23 (IL-23)	MMP-1	Interstitial collagenase	SCF	CD40 ligand (CD40-L)
IL-28a	Interferon lambda-2 (IFN-λ2)	MMP-2	72 kDa type IV collagenase	SDF-1-α+β	c-kit ligand
IL-29/IFN-λ1	Interferon lambda-1 (IFN-λ1)	MMP-3	Stromelysin-1 (SL-1)	sEGFR	Stromal cell-derived factor 1 alpha and beta (SDFA a-1-α and ß)
IL-33	Interleukin-33 (IL-33)	MMP-7	Matrilysin	sFAS	Epidermal growth factor receptor
Insulin	Insulin	MMP-8	Neutrophil collagenase	sFASl	Tumor necrosis factor receptor superfamily member 6 (FASLG receptor)
IP-10	C-X-C motif chemokine 10 (CXCL10)	MMP-9	Matrix metalloproteinase-9 (MMP-9)	sgp130	Tumor necrosis factor ligand superfamily member 6 (FasL)
Leptin	Leptin	MMP-10	Stromelysin-2	sIL-1RI	Interleukin-6 receptor subunit beta (IL-6Rß)
LIF	Leukemia inhibitory factor (LIF)	MMP-12	Macrophage metalloelastase (MME)	sIL-1RII	Interleukin-1 receptor type 1 (IL-1R-1)
MCP-1	C-C motif chemokine 2 (CCL2)	MMP-13	Collagenase 3	sIL-2Ra	Interleukin-1 receptor type 2 (IL-1R-2)
MCP-2	C-C motif chemokine 8 (CCL8)	OC	Osteocalcin	sIL-4R	Interleukin-2 receptor subunit alpha (IL-2R subunit α)
MCP-3	C-C motif chemokine 7 (CCL7)	OPG	Tumor necrosis factor receptor superfamily member 11B	sIL-6R	Interleukin-4 receptor subunit alpha (IL-4R subunit α)
SOST	Interleukin-6 receptor (subunit alpha and beta) [IL-6R-(α+ß)]	TGFβ-1	Transforming growth factor beta-1 (TGF-ß-1)	TRAIL	Tumor necrosis factor ligand superfamily member 10 (TNFSF10)
sRAGE	Advanced glycosylation end product-specific receptor	TGFβ-2	Transforming growth factor beta-2 (TGF-ß-2)	TSLP	Thymic stromal lymphopoietin
sTNFRI	Tumor necrosis factor receptor superfamily member 1A (TNF-R1)	TGFβ-3	Transforming growth factor beta-3 (TGF-ß-3)	VEGF	Vascular endothelial growth factor A (VEGF-A)
sTNFRII	Tumor necrosis factor receptor superfamily member 1B (TNF-R2)	TIMP-1	Metalloproteinase inhibitor 1	VEGF-A	Vascular endothelial growth factor A (VEGF-A)
sVEGFR1	Vascular endothelial growth factor receptor 1 (VEGFR-1)	TIMP-2	Metalloproteinase inhibitor 2	VEGF-C	Vascular endothelial growth factor C (VEGF-C)
sVEGFR2	Vascular endothelial growth factor receptor 2 (VEGFR-2)	TNF-α	Tumor necrosis factor	VEGF-D	Vascular endothelial growth factor D (VEGF-D)

sVEGFR3	Vascular endothelial growth factor receptor 3 (VEGFR-3)	TNF-β	Lymphotoxin-alpha (LT-α)	Vitamin D BP	Vitamin D-binding protein (DBP)
TARC	C-C motif chemokine 17 (CCL17)	Total PSA	Total Prostate-specific antigen (Total PSA)	Vitronectin	Vitronectin (VN)
TGF-α	Transforming growth factor alpha (TGF-α)	TPO	Thyroid peroxidase (TPO)	XCL1/ Lymphotactin	Lymphotactin
